# Examining early learners’ perceptions of inclusion: adaptation of the student version of the perceptions of inclusion questionnaire for first- and second-grade students (PIQ-EARLY)

**DOI:** 10.3389/fpsyg.2023.1181546

**Published:** 2023-06-12

**Authors:** Sandra Grüter, Janka Goldan, Carmen L. A. Zurbriggen

**Affiliations:** ^1^Faculty of Educational Science, Bielefeld University, Bielefeld, Germany; ^2^Department of Special Education, Faculty of Humanities, University of Fribourg, Fribourg, Switzerland

**Keywords:** emotional inclusion, social inclusion, academic self-concept, inclusive education, measurement invariance, validity study, perceptions of inclusion questionnaire

## Abstract

Promoting the emotional and social development of students with and without special needs is a central goal of implementing inclusive education in the school system. The entry into school, and thus into the formal education system, is accompanied by emotions and changes in self-image and social relationships. For assessing emotional inclusion, social inclusion, and academic self-concept, the Perceptions of Inclusion Questionnaire (PIQ) is a widely used instrument. To date, the paper-pencil questionnaire has been used from third through ninth grades but has not yet been used with younger ages. This paper presents an adapted version of the PIQ for first- and second-grade students, which was used on two measurement time occasions (T1, *N* = 407, *M*_Age_ = 7.2; T2, *N* = 613, *M*_Age_ = 7.6). Information on students’ reading and listening comprehension was collected from the class teachers to verify whether the adapted questionnaire can be used for all students with different levels of language competencies. Measurement invariance was demonstrated to be at least scalar for all groups considered in the analyses. Students with higher rankings of reading and listening comprehension skills reported significant higher levels of emotional inclusion and academic self-concept while there were no significant differences in social inclusion. The findings suggest that the PIQ-EARLY is a suitable instrument for assessing self-perceived inclusion in first- and second-grade students. The results also highlight the importance of students’ language competencies for adjustment to school in early school years.

## 1. Introduction

The transition from early childhood education to the first year of school involves intense emotions, as well as changes in social relationships and self-concepts, and is, therefore, one of the major challenges for young students (Dockett and Perry, 2007, 2013; Perry et al., 2014). For children with special educational needs (SEN) in particular, the transition to inclusive primary schools can be difficult because of a higher risk for early school difficulties and social exclusion (McIntyre et al., 2006; Walker et al., 2012; Schwab, 2015; Marsh et al., 2017). Inclusive schools need to support the transition process to reduce transition-related stress that can lead to social–emotional problems.

Since the ratification of the UN Convention on the Rights of Persons with Disabilities (UNCRPD), inclusive education has become a legal right in more than 180 countries and is intended to provide all students with equal opportunities to participate in the education system (United Nations, 2006). Although the discussion often focuses on students with SEN, the concept of inclusion also refers to other disadvantaging factors, such as students with a first language other than the language of instruction (Kast and Schwab, 2023). The global goal implies „the presence, participation and achievement of all students vulnerable to exclusionary pressures, not only those with impairments or those who are categorised as ‘having special educational needs’” (Ainscow et al., 2013). This means not only to physically place students with different learning requirements in mainstream classroom environments and provide individualized learning opportunities that ensure the best and most appropriate support for the academic success of all students (e.g., in terms of learning development and the attainment of academic certificates) but also to address social–emotional aspects that enhance participation and life satisfaction and, at the same time, affect the educational and academic success (Ruijs and Peetsma, 2009; Hascher, 2012).

Emotional well-being in school, social participation, and academic self-concept are considered key indicators for evaluating inclusive learning settings (Zurbriggen et al., 2019). According to Hascher (2012), (emotional) well-being in school refers to the subjective feelings and attitudes of students toward school, including happiness and enjoyment, that contribute to their physical and psychological health and development, and is connected to learning. Achieving social participation is another key factor in preventing, for example, academic underachievement, school drop-out and health problems, and is, hence, closely linked to subjective well-being in school (deLara, 2019; Goldan et al., 2022). According to Koster et al. (2009), social participation of students comprises positive contact and interaction with their peers, acceptance by classmates, social relationships and friendships, as well as the students’ perception of being accepted by their peers. Various studies indicate that students with SEN are at risk of participating less and experiencing lower levels of well-being than their peers without SEN. Moreover, the academic self-concept of the students (i.e., their perceived academic proficiency) is an important outcome of education, which has an impact on students’ learning development (e.g., Möller et al., 2009) and is likewise less developed among students with SEN in inclusive classrooms compared to peers without SEN (Bear et al., 2002; Goldan et al., 2021). Screening procedures can be employed to identify problems in students’ adjustment to school at an early stage and to enable interventions by school personal and parents.

A range of instruments has been developed to evaluate the perspectives of parents or teachers to assess different facets of the social and emotional development of students in their early school years. Most of these instruments focus on social and emotional problems and behavioral issues in the context of school readiness (Darling-Churchill et al., 2015; Vaknin-Nusbaum and Tuckwiller, 2023). In recent decades, however, the concept of school readiness has been questioned, and schools nowadays are required to adapt to students’ individual transition processes and learning conditions. Measurement instruments that capture student’s perspectives on school experiences and motivational-affective constructs are essential because they involve students as experts for their own academic well-being (Kellett and Ding, 2004; Kunter and Baumert, 2006; Huebner and Furlong, 2016; de Leeuw et al., 2018). In this regard, Venetz et al. (2019) investigated the consistency between self-reports and teacher ratings on their students’ emotional and social inclusion as well as their academic self-concept and demonstrated that teachers tend to be less accurate in evaluating the psychological outcomes of their students than in assessing academic-related constructs (Venetz et al., 2019). Schwab et al. (2020), who investigated the agreement of student, parent, and teacher ratings on the same constructs, found that primary students’ self-perceived inclusion and parent ratings on their child’s inclusion correlate more strongly than the ratings of students and teachers but still differ. In light of their findings, the authors discussed that students share their emotional and social well-being and the perceptions of their academic skills with parents and teachers only to some extent, and researchers should, therefore, take self-reports into account.

While some self-assessment scales for emotional and social constructs have already been used with students aged eight onwards (e.g., Self-Description Questionnaire; Marsh, 1994) or for early adolescents from eleven onwards (e.g., Strengths and Difficulties Questionnaire, Goodman et al., 1998; Student Subjective Well-being Questionnaire; Renshaw et al., 2015), few measurement instruments can be used with grade-one or grade-two students. A particular challenge is caused by the barely existing reading skills, the level of understanding, and, in some cases, the rather extensive instruments conflicting with the concentration capacity of young students. In German-speaking countries, the Questionnaire on Emotional and Social School Experiences of First and Second Grade Elementary School Children (Rauer and Schuck, 2004) is widely used to capture children’s perspectives on basic emotional and social experiences. The questionnaire has a nonverbal response format and can be completed in groups. The questionnaire assesses the academic self-concept, well-being in school, achievement motivation, and students’ perceptions of the social climate at school and in class. In the version for first- and second-grade students, the response format is dichotomous (*true* and *not true*), which is why differentiated answers are not possible. Hence, there is a need for instruments that researchers in the field of inclusive education and also teachers at primary schools can use to examine key aspects of their students’ self-perceived inclusion in early school years.

### 1.1. The Perceptions of inclusion questionnaire

The overview of previous studies shows that the Perceptions of Inclusion Questionnaire (PIQ; Venetz et al., 2015) has been widely applied and tested for assessing emotional inclusion, social inclusion, and academic self-concept as three central indicators of successful inclusive education (Zurbriggen et al., 2019). The development of the instrument was based on the Questionnaire for Assessing Dimensions of Integration of Students (FDI 4–6; Haeberlin et al., 1989), which was frequently used in German-speaking countries in the 1990s. With a total of 45 items, the instrument was too extensive to be used in practice, especially for students with SEN (Zurbriggen, 2021). Based on the FDI 4–6, Venetz et al. (2014), therefore, developed the PIQ, a 12-item instrument that measures the three indicators of inclusive education from students’ perspective. The questionnaire consists of four items per subscale, one of which is formulated negatively. The 4-level response format ranges from 1 (*not at all true*) to 4 (*certainly true*).

Today, the instrument is used in different languages, cultures, and groups of students (e.g., Alnahdi and Schwab, 2020; Guillemot and Hessels, 2021; Schmidt et al., 2021; DeVries et al., 2022; Zwierzchowska et al., 2022). In addition to the student version (PIQ-S), versions for teachers (PIQ-T) and parents (PIQ-P) have been developed, which in turn can be deployed to assess the three dimensions relating to students from multiple perspectives (Schwab et al., 2020). The three-factor structure of the instrument and the validity of the three constructs have since been repeatedly supported in different studies (Venetz et al., 2014; DeVries et al., 2018; Guillemot and Hessels, 2021; Knickenberg et al., 2022). Furthermore, the internal consistency of the three scales can also be described as satisfactory. For example, Zurbriggen et al. (2019) found values between 0.85 and 0.94 (McDonald’s ω) among fourth-to-sixth-grade students.

Moreover, measurement invariance of the PIQ has been tested for different groups of students to allow meaningful comparisons of student groups based on responses to the questionnaire. For example, Venetz et al. (2014) identified measurement invariance for students with and without behavioral problems and with and without a migrant background. Knickenberg et al. (2020) examined the student version’s measurement invariance for students with SEN in the area of learning (SEN-L) attending inclusive schools and special education schools. They found partial measurement equivalence, as one item of the academic self-concept scale showed different understandings by the two groups. Furthermore, the instrument has been tested for measurement invariance across genders (DeVries et al., 2018; Guillemot and Hessels, 2021). In conclusion, the PIQ is applicable to children from third to ninth grades and has been translated into different languages. Nevertheless, while the teacher version of the subscale on social inclusion has already been used in research on younger children (Herrmann et al., 2021), the student version has not yet been adapted for younger students.

To include children as informants about their perspectives, researchers need to adapt their methods and instruments (Kellett and Ding, 2004). A major challenge in surveying new school entrants and children with SEN is the absence of reading literacy. Even for short questionnaires, children must be able to read and understand the items and the response format. One approach that researchers from different domains have developed recently to encourage younger children and people with intellectual disabilities to answer paper-pencil questionnaires is the use of emoji scales (Phan et al., 2019; Nicolaidis et al., 2020). For example, Wild et al. (2017) collected data on well-being in school and self-concept among students with SEN-L in small groups using a 4-level response format with emojis under close supervision with test conductors. Massey (2021) also used emoji scales in self-completion questionnaires to investigate achievement-related attitudes in eight-to-nine-year-old children. The author concluded that the use of Emojis provides opportunities to involve children as a reliable source of information in studies to capture their perspectives and attitudes toward various concepts in the context of education.

In addition to the lack of reading skills, negatively formulated items in questionnaires can cause challenges for younger students (Goldan and Schwab, 2018; Schwab et al., 2020) and students with SEN (Knickenberg et al., 2020). In the study of Knickenberg et al. (2020), who used the PIQ-S to compare educational settings for students with SEN-L, the negatively worded items showed common variance in addition to the presumed three latent factors. The authors added a methodological factor to the measurement model and recommended a revision of the items for further research using the PIQ in studies with students with SEN-L.

### 1.2. The present study

Against this background, we developed the PIQ-EARLY, an adapted version of the PIQ-S for children in first or second grades who are beginners in reading. With this adaptation, we intend to reduce language barriers and complexity regarding the answer format so that the questionnaire can be used to assess emotional inclusion (EMI), social inclusion (SOI), and academic self-concept (ASC) in the early school years. The aim of the present study is to examine the psychometric properties of the PIQ-EARLY for first- and second-grade students with different levels of literacy competencies. More specifically, we examine the postulated factorial structure and the internal consistency for the three subscales of EMI, SOI, and ASC for the total sample on two measurement occasions. For comparing the self-reported inclusion of different student groups, the adapted PIQ-EARLY has to be validated and tested for measurement invariance (Salomo and Goldan, 2022). Hence, the instrument is tested for measurement equivalence (a) for first and second graders and (b) for students with high, moderate, and low levels of competencies in reading and listening comprehension. There are limited findings regarding the effects of language competencies on the validity of survey instruments on psychosocial student outcomes. However, we anticipate that the PIQ-EARLY will perform similarly to previous studies with older students. We expect that the analyses will confirm the three factors (EMI, SOI and ASC) for the subsamples of first and second grade students and for students with different levels of listening and reading comprehension. Moreover, we expect that the PIQ-EARLY can be used independently of the students’ grade level and listening and reading comprehension skills.

After testing for scalar measurement invariance, mean differences were examined for the respective student groups. Given the fact that language skills are an important condition for social participation (Troesch et al., 2016), students with less developed language skills are a target group for inclusive education (Kast and Schwab, 2023). The aim of our study was to find out whether self-reported inclusion differs among students with different levels of language and literacy skills. A meta-analysis by Bücker et al. (2018) revealed that although students’ well-being is associated with achievement, low or high achieving students do not necessarily report low or high levels of well-being. Most of the previous studies using the PIQ-S compared students with and without SEN. For example, Alnahdi et al. (2022) implied the Arabic version of the Perceptions of Inclusion Questionnaire (PIQ-S-AR) and found group differences in the self-perceived inclusion for all three dimensions, whereas students with SEN scored lower in each dimension, noting the largest difference in ASC. In the study of DeVries et al. (2022) on a Swedish sample, the differences of students with SEN for EMI and ASC were significantly lower than for students without SEN, but not with regard to SOI. Although these studies help to understand the socio-emotional status of students with SEN in inclusive education, they do not include measurements for learning outcomes such as language competencies, which are usually lower for students with SEN, especially for SEN-L. Zurbriggen et al. (2019) included standardized mathematics and German language tests in a validation of the PIQ-S and found moderate relations of both domains with ASC, but not with SOI and EMI. However, like all existing studies that use the PIQ, this study examined older students (fourth through seventh grade). For elementary school settings, Chapman et al. (2000) found that reading-related skills of school starters predict the development of ASC. Moreover, Boyes et al. (2018) reported a link between reading difficulties and internalizing symptoms in primary school and the findings of the meta-analysis of Troesch et al. (2016) support the importance of oral skills for social participation. Against this background, we hypothesize that students with lower levels of reading comprehension skills report lower levels of EM and ASC and students with lower levels of listening comprehension skills report lower levels of SOI.

## 2. Materials and methods

### 2.1. Study design

The survey was conducted in the school year 2021–2022 in eight inclusive primary schools and one special school in North Rhine-Westphalia (Germany). The survey of students, parents, and teachers was conducted in the context of an evaluation study that focused on a new model of school assistance. The respective schools were encouraged by the district administration to participate in the study. Nevertheless, the participation for teachers, students and parents was voluntary. For the first measurement point, the response rate was about 40 percent, for the second about 60 percent. The first- and second-grade students completed the paper-and-pencil questionnaire the first time about 2 months after the start of the school year (T1) and the second time 1 month before the end of the school year (T2). The surveys were instructed by trained test conductors. Data collection, including preparation, took about one school lesson per class and per measurement occasion. The test conductors introduced themselves to the class and handed out the short questionnaire to all participating students. The students without parental permission for participation were assigned to alternative tasks (e.g., coloring pictures). After the students had entered their names on the questionnaire, the response format was explained with an example. The test conductors read the items aloud one by one and repeated them as necessary. After all students had marked their answers for one item, the test administrators read the next item. The class teacher was also present during the data collection. Precise instructions ensured that the data collection process was standardized. All interruptions and inconsistencies were documented carefully to exclude disturbing factors. The class teachers were also asked to complete a questionnaire about every student participating in the study. Moreover, parents answered a short questionnaire including socio-demographic variables.

### 2.2. Sample

The sample at T1 consists of *N* = 407 students (53.6% female, 46.4% male, *M*_age_ = 7.2 years, *SD*_age_ = 0.64) from 43 classes. Specifically, 46.9% of students attended the first grade; 50.1% were in the second grade, and 2.9% were taught in multi-age classrooms. For 46 students (11.3%), SEN were indicated by the teachers. Information on the country of birth is available for 395 students, of whom 366 (89.5%) were born in Germany and 29 (7.1%) were born in other countries (e.g., Syria). Out of the 396 parents who provided information on their country of birth, 103 (26.0%) answered that at least one parent was born in another country then Germany.

At T2, *N* = 613 students (53.5% female, 46.5% male, *M*_age_ = 7.6 years, *SD*_Age_ = 0.65) from 49 classes participated. Specifically, 47.3% of students were in the first grade; 49.1% were in second grade, and 3.6% were taught in multi-age classrooms. SEN were indicated for 83 students (13.5%). Information on the country of birth is available for 538 of the students at T2, of whom 499 (92.8%) were born in Germany and 39 (7.2%) were born in other countries. Out of the 540 parents who provided information on their country of birth, 150 (27.8%) answered that at least one parent was born in another country then Germany. The sample and the number of classes at T2 were larger because one school with six classes was not able to participate in the study at T1 due to pandemic restrictions. To ensure that the results were not biased by the modification of the sample, we tested for longitudinal measurement invariance. Since the test suggested complete measurement invariance (Supplementary Tables S1, S2), the entire sample for T2 was included in the analyses.

### 2.3. Measures

We assessed the students’ self-perceived inclusion with an adapted version of the German PIQ-S, including the subscales EMI (e.g., “I like going to school”), SOI (e.g., “I have very good relationships with my classmates”), and ASC (e.g., “I do well in my schoolwork”; Venetz et al., 2014).^1^ Emojis were used to symbolize the response options (1 = *not at all true*, 2 = *rather not true*, 3 = *somewhat true*, 4 = *certainly true*) to address the mostly low reading competencies of first and second graders. Additionally, we reformulated the negatively worded items to reduce the instrument’s complexity and prevent comprehension problems. The aim of the reformulation was to remain as close as possible to the original negative items in the wording of the items and to cover the intended construct as well as possible. For EMI, the item “I have no desire to go to school” was replaced with the positive item “I am always happy to go to school.” For SOI, we used the item “I really like being with the other kids in my class” instead of “I feel alone in my class.” Finally, for ASC, we used the item “Many things in school are easy for me” instead of “Many things in school are too difficult for me.” The adapted version of the questionnaire is available for download.^2^ At T2, the questionnaire for the class teachers included two items to assess students’ reading comprehension skills (“How well do you consider the student’s language comprehension in terms of reading?”) and listening comprehension skills (“How well do you consider the student’s language comprehension in terms of listening?”) on a 7-point scale ranging from *very poor* to *very good*. Group comparisons demonstrated significant lower levels for reading and listening comprehensions skills for students with a country of birth other than Germany (reading comprehension: *t* (389) = 3.358, *p* < 0.001; listening comprehension: *t* (388) = 7.432, *p <* 0.001) and for students with at least one parent not born in Germany (reading comprehension: *t*(390) = 3.331, *p* < 0.001; listening comprehension: *t*(389) = 6.537, *p* < 0.001).

### 2.4. Statistical analyses

In the first step, we examined the factor structure using confirmatory factor analysis (CFA) to test whether the assumed three-factor solution adequately fits the data. To assess the model fit, we used *χ*^2^ tests as well as sample-size-independent goodness-of-fit indices. According to Hu and Bentler (1999), a non-significant *χ*^2^ test, a comparative fit index (CFI) and a Tucker-Lewis index (TLI) ≥ 0.95, a root means square error of approximation (RMSEA) value ≤0.06, and a standardized root mean square residual (SRMR) value ≤0.08 indicate that the model fits the observed data well, and CFI and TLI ≥ 0.90, RMSEA ≤0.08, and SRMR ≤0.10 indicate an acceptable model fit. The CFA models were compared using likelihood ratio tests.

In the second step, measurement invariance was tested with the forward approach (Dimitrov, 2010). First, we tested whether the number of factors and the patterns of factor loadings were equal across groups (i.e., configural invariance) by adding a group factor and estimating all parameters of the model freely. For the metric invariance testing, factor loadings were constrained to be equal across groups. Additionally, the indicator intercepts were constrained to be equal to test for scalar invariance. Finally, residuals for strict invariance and means for complete invariance were constrained to be equal across groups. We performed difference tests of the corrected *χ*^2^ values to compare the nested models. A significant result indicates a deviation from the measurement invariance assumption. The *χ*^2^ test is known to respond very sensitively to sample size (e.g., Marsh et al., 1988), so we additionally used sample-size-independent goodness-of-fit indices. If the additional restrictions do not lead to a substantial model fit decline, the assumption of measurement invariance can be considered permissible for the relevant level. The model fit decline was assessed with the following criteria: significant difference of corrected *χ*^2^ and − 0.01 change in CFI and TLI, paired with changes in RMSEA of 0.015 and SRMR of 0.030 for metric or 0.015 for scalar and strict invariance (Chen, 2007).

Due to the non-normal univariate and multivariate distribution of the indicators identified by significant Mardia’s test and Anderson–Darling test, we decided to use robust estimation methods for the CFAs. Simulation studies showed that weighted least squares, which are often used for categorical data, as well as MLR-CAT, require large sample sizes for each group (> 200) (Flora and Curran, 2004; Lei and Shiverdecker, 2020). The alternatively chosen robust maximum likelihood estimator (MLR) outperforms the more restrict estimators in analysis with small sample size when latent distributions are nonnormal (Li, 2016). Additionally, MLR allows the model-based estimation of the missing values. Based on the sufficient sample size, we additionally tested the CFAs for the total samples of both measurement occasions with a weighted least squares estimator (WLSMV) to confirm the factor structure (Supplementary Table S3).

Because there were only 0.5–1.5% missing values in the responses to the PIQ-S items, a model-based estimation of missing values was performed. Additional estimation of the models with the listwise exclusion of cases with missing values did not change the results substantially. Therefore, the reported results are based on the full samples. The nested data structure (students in classes) was accounted for by entering a cluster variable into the models. To evaluate the internal consistency of the subscales, we calculated Cronbach’s α and McDonald’s ω. After ensuring at least scalar measurement invariance, we performed group comparisons using *t*-test and ANOVA. The analyses were conducted using *R* version 4.0.3 with the package lavaan (version 0.6.14, Rosseel, 2012) for CFA and measurement invariance models.

## 3. Results

### 3.1. Factorial structure

A series of CFA models were used to evaluate the factor structure of the PIQ-EARLY in the samples of T1 and T2. As expected, the single-factor model (M1) showed no appropriate model fit (Table 1). Next, we tested an alternative model with EMI and SOI as one factor and ASC as a separate factor (M2). The fit indices were not appropriate either. In line with our expectations, the three-factor solution fit the data well at both T1 and T2. In addition to the global fit indices, the standardized factor loadings indicated an adequate local model fit as well (T1: EMI 0.75 ≤ *λ* ≤ 0.82; SOI 0.45 ≤ *λ* ≤ 0.68; ASC: 0.52 ≤ *λ* ≤ 0.65; T2: EMI, T2: 0.73 ≤ *λ* ≤ 0.81; SOI 0.52 ≤ λ ≤ 0.73; ASC: 0.53 ≤ *λ* ≤ 0.70; see Supplementary Tables S4, S5 for all factor loadings). The factor loadings for the three reformulated items SW2 (T1 = 0.82, T2 = 0.81), SI3 (T1 = 0.62, T2 = 0.73), and AS4 (T1 = 0.65, T2 = 0.59) were also acceptable. Therefore, the three-factor model with four indicators per factor was used in further analysis.

**Table 1:** Fit indices of the CFA models at T1 and T2.

Model	CFI^r^	TLI^r^	RMSEA^r^	SRMR^r^	*χ*^2^	df	ΔS-B *χ*^2^	Δdf
T1 (*N* = 407)								
M1	0.766	0.714	0.121	0.094	248.538***	54		
M2	0.853	0.817	0.097	0.082	179.089***	53	45.235***	1
M3	0.981	0.976	0.035	0.035	68.926^*^	51	89.613***	2
T2 (*N* = 613)								
M1	0.737	0.679	0.134	0.096	356.158***	54		
M2	0.835	0.794	0.108	0.084	246.407***	53	56.11***	1
M3	0.992	0.990	0.023	0.036	60.512	51	107.9***	2

CFI, comparative fit index; TLI, Tucker–Lewis index; RMSEA, root mean square error of approximation; SRMR, standardized root mean residual; *χ*^2^, chi-square statistics; df, degrees of freedom; ΔS-B *χ*^2^, Satorra–Bentler scaled chi-square difference; Δdf, difference in degrees of freedom; r, robust.

*p < 0.05, ***p ≤ 0.001.

### 3.2. Internal consistency

Table 2 shows the scale statistics for both measurement occasions. At T1, the internal consistency of the scale EMI was good, as indicated by Cronbach’s α and McDonald’s ω coefficients (*α* = 0.86, *ω* = 0.87). The internal consistency for the subscales SOI and ASC were acceptable for a short scale with four items (SOI: *α* = 0.67, *ω* = 0.68; ASC: *α* =0.67, *ω* = 0.68). The scale means were positively correlated at a moderate level (0.36 ≤ *r* ≤ 0.41). At T2, the internal consistency was acceptable to good for all three scales (EMI: *α* = 0.85, *ω* = 0.85; SOI: *α* = 0.74, *ω* = 0.75; and ASC: *α* = 0.71, *ω* = 0.74). Again, the means of the three scales were moderately positively correlated (0.37 ≤ *r* ≤ 0.43).

**Table 2:** Scale statistics, reliability coefficients, and correlations at T1 and T2.

	*M*	*SD*	Min.	Max.	*α*^1^	*ω*^1^	(1)	(2)
T1								
EMI	3.55	0.66	1	4	0.86 [0.84; 0.88]	0.87 [0.83;0.90]		
SOI	3.57	0.49	1.25	4	0.67 [0.62; 0.72]	0.68 [0.62;0.75]	0.36***	.
ASC	3.45	0.55	1	4	0.67 [0.60; 0.71]	0.68 [0.61;0.74]	0.41***	0.40***
T2								
EMI	3.44	0.68	1	4	0.85 [0.83; 0.87]	85 [0.83;0.89]		
SOI	3.52	0.54	1	4	0.74 [0.70; 0.77]	0.75 [0.70;0.80]	0.39***	
ASC	3.44	0.59	1	4	0.71 [0.67; 0.75]	0.74 [0.69;0.78]	0.43***	0.37***

*N* = 407; M, mean value; SD, standard deviation; Min., Minimum; Max., Maximum; *α*, Cronbach’s alpha; *ω*, McDonald’s omega; ^1^, 95% confidence intervals.

***p ≤ 0.001.

### 3.3. Measurement invariance and mean differences across grades

To establish measurement invariance (MI) across grades, we tested the M3 model (the baseline model) in the subsamples of first- and second-grade students. Both at T1 and T2, the baseline models for first- and second-grade students demonstrated a good fit to the data. The stepwise inclusion of restrictions to test for configural, metric, scalar, strict, and complete MI did not substantially change the model fit indices for the sample at T1 (Table 3), thus indicating complete MI.

**Table 3:** Fit statistics for the measurement invariance tests across first- and second-grade students at T1.

Model	CFI^r^	ΔCFI	TLI^r^	ΔTLI	RMSEA^r^	ΔRMSEA^r^	SRMR^r^	ΔSRMR^r^	*χ*^2^/df	ΔS-B*χ*^2^	Δdf
M3 first-grade students (*n* = 191)	0.977	–	0.971	–	0.038	–	0.053	–	63.148/51	–	–
M3 second-grade students (*n* = 204)	1.000	–	1.003	–	0.000	–	0.045	–	58.350/51	–	–
Configural MI	0.991	–	0.988	–	0.025	–	0.049	–	121.596/102	–	–
Metric MI	0.988	−0.003	0.985	−0.003	0.028	+0.003	0.065	+0.016	130.464/111	9.5433	9
Scalar MI	0.985	−0.003	0.983	−0.002	0.030	+0.002	0.067	+0.002	142.281/120	12.193^.^	9
Strict MI	0.988	+0.003	0.988	+0.005	0.025	−0.005	0.070	+0.003	147.893/132	8.839	12
Complete MI	0.986	−0.002	0.987	−0.001	0.027	+0.002	0.072	+0.002	152.302/135	4.7762	3

CFI, comparative fit index; TLI, Tucker–Lewis index; RMSEA, root mean square error of approximation; SRMR, standardized root mean residual; *χ*^2^, chi-square statistics; df, degrees of freedom; ΔS-B *χ*^2^, Satorra–Bentler scaled chi-square difference; Δ, difference; r, robust.

At T2, a significant change in the *χ*^2^ value occurred when the factor loadings were set to be equal while the cut-off values for the change in CFI, SRMR, and RMSEA were not exceeded (Table 4). After we constrained the residuals to be equal to test for strict invariance, the model was rejected by the positive *χ*^2^ test and substantially changed fit indices. We concluded that the assumption of scalar invariance, but not the assumption of strict invariance, was tenable at T2 across groups of first- and second-grade students.

**Table 4:** Fit statistics for the measurement invariance tests across first- and second-grade students at T2.

Model	CFI^r^	ΔCFI	TLI^r^	ΔTLI	RMSEA^r^	ΔRMSEA^r^	SRMR^r^	ΔSRMR^r^	*χ*^2^/df	ΔS-B *χ*^2^	Δdf
M3 first-grade students (*n* = 290)	0.976	–	0.970	–	0.041	–	0.048	–	64.401/51	–	–
M3 second-grade students (*n* = 301)	0.953	–	0.939	–	0.065	–	0.048	–	98.193***/51	–	–
Configural MI	0.963	–	0.952	–	0.055	–	0.048	–	160.101***/102	–	–
Metric MI	0.953	−0.010	0.944	−0.08	0.060	+0.005	0.059	+0.011	186.281***/111	26.636**	9
Scalar MI	0.951	−0.002	0.946	+0.002	0.058	−0.002	0.060	+0.001	199.807***/120	13.059	9
Strict MI	0.918	−0.033	0.918	−0.028	0.072	+0.014	0.065	+0.005	264.108***/132	57.472***	12
Complete MI	0.919	+0.001	0.921	+0.003	0.071	−0.001	0.065	0	265.338***/135	0.50861	3

CFI, comparative fit index; TLI, Tucker–Lewis index; RMSEA, root mean square error of approximation; SRMR, standardized root mean residual; *χ*^2^, chi-square statistics; df, degrees of freedom; ΔS-B *χ*^2^, Satorra–Bentler scaled chi-square difference; Δ, difference; r, robust.

**p < 0.01, ***p ≤ 0.001.

After confirming at least scalar measurement invariance for both measurement occasions, we compared the scale means for the subsamples of first and second graders. The means and standard deviations for the three dimensions are represented in Table 5. The comparison shows that the EMI, SOI, and ASC do not differ significantly between first- and second-grade students.

**Table 5:** Results of means comparisons.

	EMI		SOI		ASC	
	*M (SD)*	*p*	*M (SD)*	*p*	*M (SD)*	*p*
T1						
First-grade students (*n* = 191)	3.61 (0.59)		3.56 (0.50)		3.46 (0.59)	
Second-grade students (*n* = 204)	3.50 (0.71)	0.074 (0.072)	3.58 (0.48)	0.697 (0.697)	3.46 (0.52)	0.949 (0.949)
T2						
First-grade students (*n* = 290)	3.47 (0.68)		3.54 (0.56)		3.46 (0.62)	
Second-grade students (*n* = 301)	3.44 (0.68)	0.619 (0.619)	3.50 (0.52)	0.419 (0.420)	3.43 (0.57)	0.560 (0.560)
Students with low reading comprehension (1–4; *n* = 139)	3.31 (0.81)		3.46 (0.57)		3.20 (0.70)	
Students with moderate reading comprehension (5–6; *n* = 195)	3.37 (0.68)		3.55 (0.48)		3.45 (0.55)	
Students with high reading comprehension (7; *n* = 106)	3.59 (0.53)	**0.006 (0.001)**	3.50 (0.49)	0.268 (0.279)	3.61 (0.50)	**<0.001 (<0.001**)
Students with low listening comprehension (1–4; *n* = 91)	3.28 (0.86)		3.43 (0.61)		3.12 (0.77)	
Students with moderate listening comprehension (5–6; *n* = 216)	3.36 (0.70)		3.55 (0.47)		3.41 (0.56)	
Students with high listening comprehension high (7; *n* = 132)	3.54 (0.57)	0.**015 (0.009)**	3.49 (0.50)	0.130 (0.170)	3.60 (0.48)	**<0.001 (<0.001)**

*p*-values (two-sided) for *t*-test or ANOVA, *p*-values for Welch’s test in parentheses; *M*, mean value; *SD*, standard deviation; boldface indicates significant differences.

### 3.4. Measurement invariance and mean differences across students with low, moderate, and high reading comprehension skills

To examine MI with regard to students’ reading competencies, we divided the total samples at T2 into three subsamples: students with low (answer options 1–4, *n* = 139), moderate (5–6, *n* = 195), and high reading comprehension (7, *n* = 106). As a condition for equating additional parameters across the groups with different levels of reading skills, the fit of the baseline model was assessed in these subsamples. A good model fit was demonstrated for the baseline model in the subsamples with low, moderate, and high reading comprehension (Table 6). The stepwise inclusion of restrictions for the configural, metric, and scalar invariance models did not significantly change the model fit. Equating the residuals resulted in a significant chi-square test and changes in the fit indices. We concluded that the assumption of scalar measurement invariance, but not the assumption of strict invariance, was tenable across the groups.

**Table 6:** Fit Statistics for the measurement invariance tests across students with low, moderate, and high reading comprehension skills at T2.

Model	CFI^r^	ΔCFI	TLI^r^	ΔTLI	RMSEA^r^	ΔRMSEA^r^	SRMR^r^	ΔSRMR^r^	*χ*^2^/df	ΔS-B *χ*^2^	Δdf
M3 students with low reading comprehension (1–4; *n* = 139)	0.928	–	0.906	–	0.067	–	0.066	–	71.979^*^/51	–	–
M3 students with moderate reading comprehension (5–6; *n* = 195)	0.983	–	0.978	–	0.037	–	0.060	–	64.606/51	–	–
M3 students with high reading comprehension (7; *n* = 106)	0.958	–	0.945	–	0.054	–	0.078	–	77.831^**^/51	–	–
Configural MI	0.962	–	0.951	–	0.052	–	0.066	–	213.445***/153	–	–
Metric MI	0.950	−0.012	0.942	−0.009	0.057	+0.005	0.082	+0.016	237.846***/171	24.582	18
Scalar MI	0.948	−0.002	0.945	+0.003	0.055	−0.002	0.084	+0.002	260.710***/189	22.259	18
Strict MI	0.804	−0.144	0.818	−0.127	0.100	+0.045	0.108	+0.024	436.933***/213	146.93***	24
Complete MI	0.781	−0.023	0.802	−0.016	0.105	+0.005	0.122	+0.014	470.165***/219	37.014***	6

CFI, comparative fit index; TLI, Tucker–Lewis index; RMSEA, root mean square error of approximation; SRMR, standardized root mean residual; *χ*^2^, chi-square statistics; df, degrees of freedom; ΔS-B *χ*^2^, Satorra–Bentler scaled chi-square difference; Δ, difference; r, robust.

*p < 0.05, **p < 0.01, ***p ≤ 0.001.

Comparing students with low, moderate, and high reading comprehension skills revealed significant differences in EMI and ASC (Table 5). The group mean differences indicate lower scores for the group of students with low reading skills compared to their peers with moderate and high reading skills. *Post hoc* tests (Bonferroni corrected) indicated significant differences in EMI comparing students with low versus high reading skills and comparing students with moderate versus high reading skills.

### 3.5. Measurement invariance across students with low, moderate, and high listening comprehension skills

To examine MI with regard to listening comprehension skills, we divided the total samples at T2 into three subsamples: students with low (answer options 1–4, *n* = 91), moderate (5–6, *n* = 216), and listening comprehension (7, *n* = 132). As a condition for equating additional parameters across the groups with different levels of reading skills, the fit of the baseline model was assessed in these subsamples.

A good model fit was demonstrated for the baseline model (Table 7), and the assumption for configural invariance was confirmed. Although the stepwise inclusion of restrictions for the metric and scalar invariance model led to a positive *χ*^2^ difference test, the changes in fit indices were still acceptable. Equating the residuals in the strict invariance model resulted in a significant *χ*^2^ test and substantial changes in the fit indices. We concluded that the assumption of scalar measurement invariance, but not the assumption of strict invariance, was tenable across the groups.

**Table 7:** Fit statistics of the measurement invariance tests across students with low, moderate, and high listening comprehension skills at T2.

Model	CFI^r^	ΔCFI	TLI^r^	ΔTLI	RMSEA^r^	ΔRMSEA^r^	SRMR^r^	ΔSRMR^r^	*χ*^2^/df	ΔS-B *χ*^2^	Δdf
M3 students with low listening comprehension (1–4; *n* = 91)	1.000	–	1.050	–	0.000	–	0.064	–	45.628/51	–	–
M3 students with moderate listening comprehension (5–6; *n* = 216)	1.000	–	1.000	–	0.005	–	0.052	–	55.307/51	–	–
M3 students with high listening comprehension (7; *n* = 132)	0.969	–	0.960	–	0.049	–	0.066	–	69.240*/51	–	–
Configural MI	0.996	–	0.995	–	0.017	–	0.059	–	170.185/153	–	–
Metric MI	0.979	−0.017	0.976	−0.019	0.037	+0.020	0.078	+0.019	202.187/171	29.884*	18
Scalar MI	0.969	−0.010	0.968	−0.008	0.043	+0.006	0.083	+0.005	234.511*/189	35.893**	18
Strict MI	0.766	−0.203	0.783	−0.185	0.110	+0.067	0.115	+0.032	480.488***/213	217.45***	24
Complete MI	0.745	−0.021	0.769	−0.014	0.113	+0.003	0.128	+0.013	514.766***/219	43.019***	6

CFI, comparative fit index; TLI, Tucker–Lewis index; RMSEA, root mean square error of approximation; SRMR, standardized root mean residual; *χ*^2^, chi-square statistics; df, degrees of freedom; ΔS-B *χ*, Satorra–Bentler scaled chi-square difference; Δ, difference; r, robust.

*p < 0.05, **p < 0.01, ***p ≤ 0.001.

Comparing the means for the subsamples of students with high, moderate, and low competencies in listening comprehension revealed significant differences in EMI and ASC. *Post hoc* tests (Bonferroni corrected) indicated significant differences in EMI when comparing students with low versus high reading skills (Table 5). For ASC, all pairwise group comparisons showed significant group differences (low vs. moderate, moderate vs. high, and low vs. high listening comprehension). There were no significant group differences in SOI.

## 4. Discussion

Inclusive education aims to provide individual learning opportunities and positive emotional and social experiences for all students vulnerable to exclusionary pressures (Ainscow et al., 2013), which includes students with less developed language skills in the language of instruction (Kast and Schwab, 2023). In particular, the period of transition from early childhood education to primary school is accompanied by changes in the academic demands that affect the students’ social relationships, motivation and emotions (Griebel and Niesel, 2002; Walker et al., 2012). Measurement instruments that capture self-perceived inclusion are useful not only in school practice but also in research on the effects of inclusive schooling on socio-emotional outcomes. The student version of the PIQ is a well-established questionnaire for student self-reports (Venetz et al., 2015; Knickenberg et al., 2022). The aim of this study was to adapt the PIQ-S for first- and second-grade students and test the new PIQ-EARLY for factor validity. Furthermore, to allow group comparisons, we tested the questionnaire for measurement invariance in first- and second-grade students and among groups of students with different levels of language competencies. Using the suggestions made by Knickenberg et al. (2020), we adjusted the negative items of the original questionnaire in a positive direction. Additionally, we added an emoji scale to symbolize the answer options. The adapted questionnaire was implemented in grades one and two at eight inclusive primary schools and one special school on two measurement occasions.

Findings support the three-factor structure of the PIQ-EARLY with the three dimensions: emotional inclusion, social inclusion and academic self-concept. The indices indicate at least an acceptable model fit for the baseline model within the complete sample and within the subsamples for both measurement occasions. All items, including the adapted items, showed substantial and significant factor loadings. After confirming the baseline model in the considered subsamples, we also tested for measurement invariance. The results indicate that the questionnaire items were equally understood by the groups of first- and second-grade students and students with different levels of reading and listening comprehension skills. Therefore, it is possible to interpret mean differences between the groups.

In line with our expectations, the results of the group comparisons demonstrate higher levels of emotional inclusion and academic self-concept for students with highly developed reading comprehension skills in comparison to the groups of students with lower reading skills. The same pattern was found for listening comprehension. These results replicate the findings of prior studies using the PIQ-S for comparisons of students with and without SEN (DeVries et al., 2022). Consistent with our findings, Zurbriggen et al. (2019) found a relationship between academic self-concept and competencies in the language of instruction in older school children. However, in contrast to the present study, they found no significant correlation of achievement tests in German and emotional inclusion. The emotional well-being of students’ may be more dependent on language skills at the early beginning of schooling, because students have to learn important academic skills including reading in this critical phase (Vaknin-Nusbaum and Tuckwiller, 2023), and adjustment to school is especially challenging when students do not understand the classroom language. These findings highlight the relevance of early language support before the start of primary school, especially for children with a less supportive literacy environment at home (Ebert et al., 2020). Although language competencies are a critical foundation for social relationships (Troesch et al., 2016), no significant differences in social inclusion were found between the compared groups. To further examine the relationships between language skills and social participation, future analyses should include class composition and students’ first language.

The current study has several limitations that should be considered when interpreting the results. First, the sample of the first measurement occasion is smaller than the sample of the second. Although the constraints caused by the COVID-19 pandemic explain this fact, equally sized samples and longitudinal data for all students would be beneficial for analyzing invariance across measurement occasions. Furthermore, the subsample of students with SEN was too small to test for measurement invariance. Although Knickenberg et al. (2020) validated the PIQ-S on a sample of students with 8- to 15-year-old SEN-L, a replication for primary students with SEN with the adapted PIQ-EARLY would be advisable for the use of the questionnaire in inclusive primary schools and special schools.

Finally, the data on reading and listening comprehension skills were collected from the affiliated class teachers using one item, respectively. The aim was to economically assess the students’ language competences. Based on this information, it was possible to examine differences in emotional and social school experiences that are related to students’ language skills at the beginning of schooling. However, due to the lack of objectivity in evaluating the students’ performance by a one-item measure from the teachers’ perspective, academic performance data on language competencies from standardized tests would be a more reliable source. Various tests are available that can be used to identify children with lower language and literacy skills. Test selection should take the age level, theoretical framework, and domains of the subtest into account. The study by Keenan and Meenan (2014) showed that four comparative reading comprehension tests identify different school children as needing support. Therefore, researchers should use established test procedures that fit the research objective. In future studies, the widely used ELFE test could be used as a tool for assessing reading comprehension in German (Lenhard et al., 2018). Language comprehension can also be assessed as part of broader testing procedures of fundamental cognitive competencies of preschool children (e.g., BASIC-PRESCHOOL; Daseking and Petermann, 2008). In the US, for example, the Phonological Awareness Literacy Screening for Preschoolers (PALS-PreK; Invernizzi et al., 2004) was designed to measure children’s literacy skills in 4-year-olds. If researchers are interested in examining differences between groups of older students with different academic performance levels, student grades in different subjects could also be used as indicators.

In conclusion, the PIQ-EARLY can be applied in the first years of primary school to assess students’ self-reports on their emotional and social inclusion, and academic self-concept as three central indicators for successful inclusion in the education system. Moreover, the results of the measurement invariance test showed that the questionnaire performs independently of the grade and students’ language competencies. Therefore, the adapted questionnaire can be used in further investigations and in school life to involve students as experts for their well-being, especially during the transition process.

## Data availability statement

The original contributions presented in the study are included in the article/Supplementary material, further inquiries can be directed to the corresponding author.

## Ethics statement

Ethical review and approval was not required for the study on human participants in accordance with the local legislation and institutional requirements. Written informed consent to participate in this study was provided by the participants’ legal guardian/next of kin.

## Author contributions

SG and JG were actively involved in the planning and implementation of the data collection, wrote the introduction and discussed it with CZ. CZ actively contributed to the adaptation of the PIQ and was a consultant to SG and JG regarding the study design and survey instruments. SG conducted and described the analyses in close consultation with CZ. All authors contributed to the article and approved the submitted version.

## Conflict of interest

The authors declare that the research was conducted in the absence of any commercial or financial relationships that could be construed as a potential conflict of interest.

## Publisher’s note

All claims expressed in this article are solely those of the authors and do not necessarily represent those of their affiliated organizations, or those of the publisher, the editors and the reviewers. Any product that may be evaluated in this article, or claim that may be made by its manufacturer, is not guaranteed or endorsed by the publisher.

## Footnotes

^1^For the wording of all 12 items, see www.piqinfo.ch

^2^https://piqinfo.ch/, since only the German version has been validated so far, the PIQ-EARLY is currently exclusively available in German.

## References

[ref1] Ainscow M. Dyson A. Weiner S. (2013). From exclusion to inclusion: ways of responding in schools to students with special educational needs. Reading: CfBT Education Trust.

[ref2] Alnahdi G. Lindner K.-T. Schwab S. (2022). Teachers’ implementation of inclusive teaching practices as a potential predictor for students’ perception of academic, social and emotional inclusion. Front. Psychol. 13:917676. doi: 10.3389/fpsyg.2022.917676, PMID: 35967702 PMC9369003

[ref3] Alnahdi G. H. Schwab S. (2020). Psychometric properties of the Arabic version of the behavioral intention to interact with peers with intellectual disability scale. Front. Psychol. 11:1212. doi: 10.3389/fpsyg.2020.01212, PMID: 32636780 PMC7317011

[ref4] Bear G. G. Minke K. M. Manning M. A. (2002). Self-concept of students with learning disabilities: a meta-analysis. Sch. Psychol. Rev. 31, 405–427. doi: 10.1080/02796015.2002.12086165

[ref5] Boyes M. Tebbutt B. Preece K. Badcock N. (2018). Relationships between Reading ability and child mental health: moderating effects of self-esteem. Aust. Psychol. 53, 125–133. doi: 10.1111/ap.12281

[ref6] Bücker S. Nuraydin S. Simonsmeier B. A. Schneider M. Luhmann M. (2018). Subjective well-being and academic achievement: a meta-analysis. J. Res. Pers. 74, 83–94. doi: 10.1016/j.jrp.2018.02.007

[ref7] Chapman J. W. Tunmer W. E. Prochnow J. E. (2000). Early reading-related skills and performance, reading self-concept, and the development of academic self-concept: a longitudinal study. J. Educ. Psychol. 92, 703–708. doi: 10.1037/0022-0663.92.4.703

[ref8] Chen F. (2007). Sensitivity of goodness of fit indexes to lack of measurement invariance. Struct. Equ. Model. Multidiscip. J. 14, 464–504. doi: 10.1080/10705510701301834

[ref9] Darling-Churchill K. Chien N. Halle T. Lippmann L. Zaslow M. Daneri P.., (2015). Characteristics of existing measures of social and emotional development in early childhood. Available at: https://www.childtrends.org/publications/characteristics-of-existing-measures-of-social-and-emotional-development-in-early-childhood

[ref10] Daseking M. Petermann F. (2008). Screening für kognitive Basiskompetenzen im Vorschulalter [screening for basic cognitive skills at preschool age] Bern Huber.

[ref11] de Leeuw R. R. de Boer A. A. Bijstra J. Minnaert A. E. M. G. (2018). Teacher strategies to support the social participation of students with SEBD in the regular classroom. Eur. J. Spec. Needs Educ. 33, 412–426. doi: 10.1080/08856257.2017.1334433

[ref12] deLara E. W. (2019). Consequences of childhood bullying on mental health and relationships for young adults. J. Child Fam. Stud. 28, 2379–2389. doi: 10.1007/s10826-018-1197-y

[ref13] DeVries J. M. Knickenberg M. Trygger M. (2022). Academic self-concept, perceptions of inclusion, special needs and gender: evidence from inclusive classes in Sweden. Eur. J. Spec. Needs Educ. 37, 511–525. doi: 10.1080/08856257.2021.1911523

[ref14] DeVries J. M. Voß S. Gebhardt M. (2018). Do learners with special education needs really feel included? Evidence from the perception of inclusion questionnaire and strengths and difficulties questionnaire. Res. Dev. Disabil. 83, 28–36. doi: 10.1016/j.ridd.2018.07.007, PMID: 30098453

[ref15] Dimitrov D. M. (2010). Testing for factorial invariance in the context of construct validation. Meas. Eval. Couns. Dev. 43, 121–149. doi: 10.1177/0748175610373459

[ref16] Dockett S. Perry B. (2007). Transitions to school: perceptions, expectations, experiences. Sydney UNSW Press.

[ref17] Dockett S. Perry B. (2013). Trends and tensions: Australian and international research about starting school. Int. J. Early Years Educ. 21, 163–177. doi: 10.1080/09669760.2013.832943

[ref18] Ebert S. Lehrl S. Weinert S. (2020). Differential effects of the home language and literacy environment on child language and theory of mind and their relation to socioeconomic background. Front. Psychol. 11:555654. doi: 10.3389/fpsyg.2020.555654, PMID: 33192809 PMC7658343

[ref19] Flora D. B. Curran P. J. (2004). An empirical evaluation of alternative methods of estimation for confirmatory factor analysis with ordinal data. Psychol. Methods 9, 466–491. doi: 10.1037/1082-989X.9.4.466, PMID: 15598100 PMC3153362

[ref20] Goldan J. Hoffmann L. Schwab S. (2021). “A matter of resources? – students’ academic self-concept, social participation and school-wellbeing in inclusive education” in International perspectives on inclusive education. eds. Goldan J. Lambrecht J. Loreman T., Resourcing Inclusive Education, vol. 15 (Bingley: Emerald), 89–100.

[ref21] Goldan J. Nusser L. Gebel M. (2022). School-related subjective well-being of children with and without special educational needs in inclusive classrooms. Child Indic. Res. 15, 1313–1337. doi: 10.1007/s12187-022-09914-8

[ref22] Goldan J. Schwab S. (2018). Measuring students’ and teachers’ perceptions of resources in inclusive education – validation of a newly developed instrument. Int. J. Incl. Educ. 24, 1326–1339. doi: 10.1080/13603116.2018.1515270

[ref23] Goodman R. Meltzer H. Bailey V. (1998). The strengths and difficulties questionnaire: a pilot study on the validity of the self-report version. Eur. Child Adolesc. Psychiatry 7, 125–130. doi: 10.1007/s007870050057, PMID: 9826298

[ref24] Griebel W. Niesel R. (2002). “Co-constructing transition into kindergarten and school by children, parents and teachers” in Transitions in the early years. Debating continuity and progression for young children in early education. eds. Fabian H. Dunlop A.-W. (London: Routledge-Falmer), 64–75.

[ref25] Guillemot F. Hessels M. G. P. (2021). Validation of the student version of the perceptions of inclusion questionnaire on a sample of French students. Eur. J. Spec. Needs Educ. 37, 850–865. doi: 10.1080/08856257.2021.1961195

[ref26] Haeberlin U. Moser U. Bless G. Klaghofer R. (1989). “Integration in die Schulklasse” in Fragebogen zur Erfassung von Dimensionen der Integration von Schülern FDI (Bern: Haupt), 4–6.

[ref27] Hascher T. (2012). “Well-being and learning in school” in Encyclopedia of the sciences of learning. ed. Seel N. M. (Heidelberg: Springer), 3453–3456.

[ref28] Herrmann C. Bretz K. Kühnis J. Seelig H. Keller R. Ferrari I. (2021). Connection between social relationships and basic motor competencies in early childhood. Children 8:53. doi: 10.3390/children8010053, PMID: 33477320 PMC7829824

[ref29] Hu L. Bentler P. M. (1999). Cut-off criteria for fit indexes in covariance structure analysis: conventional criteria versus new alternatives. Struct. Equ. Model. Multidiscip. J. 6, 1–55. doi: 10.1080/10705519909540118

[ref30] Huebner E. S. Furlong M. (2016). “Measuring students’ well-being” in Promoting student happiness: positive psychology intervention in schools. ed. Suldo S. M. (New York, NY: Guilford Press)

[ref31] Invernizzi M. Meier J. Swank L. (2004). Phonological awareness literacy screening for preschoolers (PALS-PreK). Charlottesville, University of Virginia. doi: 10.1037/t27727-000

[ref32] Kast J. Schwab S. (2023). Teachers’ and parents’ attitudes towards inclusion of pupils with a first language other than the language of instruction. Int. J. Incl. Educ. 27, 221–240. doi: 10.1080/13603116.2020.1837267

[ref33] Keenan J. M. Meenan C. (2014). Test differences in diagnosing reading comprehension deficits. J. Learn. Disabil. 47, 125–135. doi: 10.1177/0022219412439326, PMID: 22442251 PMC3383937

[ref34] Kellett M. Ding S. (2004). “Middle childhood” in Doing research with children and young people. eds. Fraser S. Lewis V. Ding S. Kellet M. Robinson C. (London: SAGE), 161–174.

[ref35] Knickenberg M. Zurbriggen C. L. A. Schwab S. (2022). Validation of the student version of the perceptions of inclusion questionnaire in primary and secondary education settings. SAGE Open 12:215824402210798. doi: 10.1177/21582440221079896

[ref36] Knickenberg M. Zurbriggen C. L. A. Venetz M. Schwab S. Gebhardt M. (2020). Assessing dimensions of inclusion from students’ perspective – measurement invariance across students with learning disabilities in different educational settings. Eur. J. Spec. Needs Educ. 35, 287–302. doi: 10.1080/08856257.2019.1646958

[ref37] Koster M. Nakken H. Pijl S. J. Van Houten E. (2009). Being part of the peer group: a literature study focusing on the social dimension of inclusion in education. Int. J. Incl. Educ. 13, 117–140. doi: 10.1080/13603110701284680

[ref38] Kunter M. Baumert J. (2006). Who is the expert? Construct and criteria validity of student and teacher ratings of instruction. Learn. Environ. Res. 9, 231–251. doi: 10.1007/s10984-006-9015-7

[ref39] Lei P.-W. Shiverdecker L. K. (2020). Performance of estimators for confirmatory factor analysis of ordinal variables with missing data. Struct. Equ. Model. 27, 584–601. doi: 10.1080/10705511.2019.1680292

[ref40] Lenhard W. Lenhard A. Schneider W. (2018). ELFE II. Ein Leseverständnistest für Erst- bis Siebtklässler-Version II. [ELFE II. A reading comprehension test for first to seventh-graders]. Göttingen: Hogrefe.

[ref41] Li C. H. (2016). Confirmatory factor analysis with ordinal data: comparing robust maximum likelihood and diagonally weighted least squares. Behav. Res. Methods 48, 936–949. doi: 10.3758/s13428-015-0619-7, PMID: 26174714

[ref42] Marsh H. W. (1994). Using the National Educational Longitudinal Study of 1988 to evaluate theoretical models of self-concept: the self description questionnaire. J. Educ. Psychol. 86, 439–456. doi: 10.1037/0022-0663.86.3.439

[ref43] Marsh H. W. Balla J. R. McDonald R. P. (1988). Goodness-of-fit indexes in confirmatory factor analysis: the effect of sample size. Psychol. Bull. 103, 391–410. doi: 10.1037//0033-2909.103.3.391

[ref44] Marsh A. Spagnol V. Grove R. Eapen V. (2017). Transition to school for children with autism spectrum disorder: a systematic review. World J. Psychiatry 7, 184–196. doi: 10.5498/wjp.v7.i3.184, PMID: 29043156 PMC5632603

[ref45] Massey S. (2021). Using Emojis and drawings in surveys to measure children’s attitudes to mathematics. Int. J. Soc. Res. Methodol. 25, 877–889. doi: 10.1080/13645579.2021.1940774

[ref47] McIntyre L. L. Blacher J. Baker B. L. (2006). The transition to school: adaptation in young children with and without intellectual disability. J. Intellect. Disabil. Res. 50, 349–361. doi: 10.1111/j.1365-2788.2006.00783.x, PMID: 16629928

[ref48] Möller J. Pohlmann B. Köller O. Marsh H. W. (2009). A Meta-analytic path analysis of the internal/external frame of reference model of academic achievement and academic self-concept. Rev. Educ. Res. 79, 1129–1167. doi: 10.3102/0034654309337522

[ref49] Nicolaidis C. Raymaker D. M. McDonald K. E. Lund E. M. Leotti S. Kapp S. K.. (2020). Creating accessible survey instruments for use with autistic adults and people with intellectual disability: lessons learned and recommendations. Autism Adulthood 2, 61–76. doi: 10.1089/aut.2019.0074, PMID: 32355908 PMC7188318

[ref50] Perry B. Dockett S. Petriwskyj A. (Eds.). (2014). Transitions to school – international research, policy and practice. Dordrecht Springer.

[ref51] Phan W. M. J. Amrhein R. Rounds J. Lewis P. (2019). Contextualizing interest scales with Emojis: implications for measurement and validity. J. Career Assess. 27, 114–133. doi: 10.1177/1069072717748647

[ref52] Rauer W. Schuck K.-D. (2004). Fragebogen zur Erfassung emotionaler und sozialer Schulerfahrungen von Grundschulkindern erster und Zweiter Klassen [Questionnaire on emotional and social School experiences of first and second grade elementary school children] Beltz.

[ref53] Renshaw T. L. Long A. C. J. Cook C. R. (2015). Assessing adolescents’ positive psychological functioning at school: development and validation of the student subjective well-being questionnaire. Sch. Psychol. Q. 30, 534–552. doi: 10.1037/spq0000088, PMID: 25180834

[ref54] Rosseel Y. (2012). Lavaan: an R package for structural equation modeling. J. Stat. Softw. 48, 1–36. doi: 10.18637/jss.v048.i02

[ref55] Ruijs N. M. Peetsma T. T. S. (2009). Effects of inclusion on students with and without special educational needs reviewed. Educ. Res. Rev. 4, 67–79. doi: 10.1016/j.edurev.2009.02.002

[ref56] Salomo K. Goldan J. (2022). “Measurement invariance” in The SAGE encyclopedia of research design. ed. Frey B. B. (Thousand Oaks, CA: SAGE)

[ref57] Schmidt M. Schwab S. Zurbriggen C. L. A. (2021). Social inclusion, emotional inclusion and academic self-concept of Slovenian students with learning disabilities. Educ. Stud. 72, 218–234. doi: 10.1080/03055698.2021.506319

[ref58] Schwab S. (2015). Social dimensions of inclusion in education of 4th and 7th grade pupils in inclusive and regular classes: outcomes from Austria. Res. Dev. Disabil. 43–44, 72–79. doi: 10.1016/j.ridd.2015.06.005, PMID: 26159883

[ref59] Schwab S. Zurbriggen C. L. A. Venetz M. (2020). Agreement among student, parent and teacher ratings of school inclusion: a multitrait-multimethod analysis. J. Sch. Psychol. 82, 1–16. doi: 10.1016/j.jsp.2020.07.003, PMID: 32988457

[ref60] Troesch L. M. Keller K. Grob A. (2016). Language competence and social preference in childhood. Eur. Psychol. 21, 167–179. doi: 10.1027/1016-9040/a000262

[ref61] United Nations (2006). Convention on the rights of persons with disabilities. Available at: https://www.un.org/development/desa/disabilities/convention-on-the-rights-of-persons-with-disabilities.html 10.1515/9783110208856.203 18348362

[ref62] Vaknin-Nusbaum V. Tuckwiller E. D. (2023). Reading motivation, well-being and reading achievement in second grade students. J. Res. Read. 46, 64–85. doi: 10.1111/1467-9817.12414

[ref63] Venetz M. Zurbriggen C. Eckhart M. (2014). Entwicklung und erste Validierung einer Kurzversion des “Fragebogens zur Erfassung von Dimensionen der integration von Schülern (FDI 4–6)” von Haeberlin, Moser, Bless und Klaghofer [development and first validation of a short version of the questionnaire for measuring dimensions of integration of students (FDI 4-6) by Haeberlin, Moser, Bless, and Klaghofer]. Empirische Sonderpädagogik 6, 99–113. doi: 10.25656/01:9247

[ref64] Venetz M. Zurbriggen C. L. A. Eckhart M. Schwab S. Hessels M. G. P. (2015). The perceptions of inclusion questionnaire (PIQ). Available at: http://www.piqinfo.ch

[ref65] Venetz M. Zurbriggen C. L. A. Schwab S. (2019). What do teachers think about their students’ inclusion? Consistency of students’ self-reports and teacher ratings. Front. Psychol. 10:1637. doi: 10.3389/fpsyg.2019.01637, PMID: 31379672 PMC6646673

[ref66] Walker S. Dunbar S. Meldrum K. Whiteford C. Carrington S. Hand K.. (2012). The transition to school of children with developmental disabilities: views of parents and teachers. Australas. J. Early Childhood 37, 22–29. doi: 10.1177/183693911203700304

[ref67] Wild E. Lütje-Klose B. Schwinger M. Gorges J. Neumann P. (2017). Bielefelder Längsschnittstudie zum Lernen in inklusiven und exklusiven Förderarrangements [Bielefeld Longitudinal Study on Learning in Inclusive and Exclusive Support Arrangements] (BiLieF). Technical Report. Bielefeld: Universität Bielefeld.

[ref68] Zurbriggen C. (2021). “Was und wie gut misst der perceptions of inclusion questionnaire? Qualität, Spezifität und (Weiter-)Entwicklung [what and how well does the perceptions of inclusion questionnaire measure? Quality, specificity and (further) development]” in Heilpädagogische Forschung: Bildung für Alle. ed. Klaver P. Interkantonale Hochschule für Heilpädagogik

[ref69] Zurbriggen C. L. A. Venetz M. Schwab S. Hessels M. G. P. (2019). A psychometric analysis of the student version of the perceptions of inclusion questionnaire (PIQ). Eur. J. Psychol. Assess. 35, 641–649. doi: 10.1027/1015-5759/a000443

[ref70] Zwierzchowska A. Kostorz K. Rosołek B. Tomińska-Conte E. (2022). Validation of the perceptions of inclusion questionnaire including PE teachers’ opinion as part of an innovative use of the tool. Sustainability 14:3241. doi: 10.3390/su14063241

