# Supplementary material for: Examining early learners’ perceptions of inclusion: adaptation of the student version of the perceptions of inclusion questionnaire for first- and second-grade students (PIQ-EARLY)
Source: Front Psychol. 2023 Jun 12;14:1181546. doi: 10.3389/fpsyg.2023.1181546 (PMC10291259; doi:10.3389/fpsyg.2023.1181546)
Supplement: Supplementary file 1 [file Table_1.pdf]

Supplementary Material

Examining Early Learners' Perceptions of Inclusion: Adaptation of the Student Version of the Perceptions of Inclusion Questionnaire for First- and Second-Grade Students (PIQ-EARLY)

Sandra Grüter*, Janka Goldan, Carmen L. A. Zurbriggen

*** Correspondence:**

Sandra Grüter:
sandra.grueter@uni-bielefeld.de

Supplementary Table 1

Fit Statistics of the Measurement Invariance Tests without School 6010

Model	CFI^r	ΔCFI	TLI^r	ΔTLI	$RMSEA^r$	$\Delta RMSEA^r$	$SRMR^r$	$\Delta SRMR^r$	χ^2/df	ΔS-B χ^2	Δdf
$M3_{T1}$ (n = 407)	0.981	-	0.976	-	0.035	-	0.035	-	68.926*/51	-	-
$M3_{T2}$ (n = 520)	0.995	-	0.994	-	0.019	-	0.036	-	57.670/51	-	-
Configural MI	0.989	-	0.986	-	0.027	-	0.035	-	125.843/102	-	-
Metric MI	0.992	+0.003	0.991	+0.005	0.023	-0.004	0.040	+0.005	128.819/111	4.301	9
Scalar MI	0.993	+0.001	0.992	+0.001	0.021	-0.002	0.040	0	137.296/120	7.6738	9
Strict MI	0.993	0	0.993	+0.001	0.019	-0.002	0.045	+0.005	147.105/132	11.066	12
Complete MI	0.993	0	0.993	0	0.020	+0.001	0.047	+0.002	151.345/135	4.4671	3

Note.
CFI = comparative fit index; TLI = Tucker–Lewis index; RMSEA = root mean square error of approximation; SRMR = standardized root mean residual; χ^2 *= chi-square statistics; df = degrees of freedom; ΔS-B* χ^2 *= Satorra–Bentler scaled chi-square difference; Δdf = difference in degrees of freedom, r = robust.*
* $p < 0.05$, ** $p < .01$, *** $p \leq .001$.
