# Supplementary material for: Examining early learners’ perceptions of inclusion: adaptation of the student version of the perceptions of inclusion questionnaire for first- and second-grade students (PIQ-EARLY)
Source: Front Psychol. 2023 Jun 12;14:1181546. doi: 10.3389/fpsyg.2023.1181546 (PMC10291259; doi:10.3389/fpsyg.2023.1181546)
Supplement: Supplementary file 2 [file Table_2.pdf]

Supplementary Material

Examining Early Learners' Perceptions of Inclusion: Adaptation of the Student Version of the Perceptions of Inclusion Questionnaire for First- and Second-Grade Students (PIQ-EARLY)

Sandra Grüter*, Janka Goldan, Carmen L. A. Zurbriggen

*** Correspondence:**

Sandra Grüter:
sandra.grueter@uni-bielefeld.de

Supplementary Table 2

Fit Statistics of the Measurement Invariance Tests with the Full Samples of T1 and T2

Model	CFI^r	ΔCFI	TLI^r	ΔTLI	$RMSEA^r$	$ΔRMSEA^r$	$SRMR^r$	$ΔSRMR^r$	χ^2/df	ΔS-B χ^2	Δdf
$M3_{T1}$ (*n* = 407)	0.981	-	0.976	-	0.035	-	0.035	-	68.926*/51	-	-
$M3_{T2}$ (*n* = 613)	0.992	-	0.990	-	0.023	-	0.036	-	60.512/51	-	-
Configural MI	0.988	-	0.985	-	0.029	-	0.035	-	128.889*/102	-	
Metric MI	0.991	+0.003	0.989	+0.004	0.024	-0.005	0.038	+0.003	131.535/111	4.3376	9
Scalar MI	0.992	+0.001	0.991	+0.002	0.022	-0.002	0.039	+0.001	138.896/120	6.0844	9
Strict MI	0.992	0	0.992	+0.001	0.021	-0.001	0.042	+0.003	149.889/132	11.938	12
Complete MI	0.991	-0.001	0.991	-0.001	0.022	+0.001	0.045	+0.003	154.514/135	4.9444	3

Note.
CFI = comparative fit index; TLI = Tucker–Lewis index; RMSEA = root mean square error of approximation; SRMR = standardized root mean residual; χ^2 = chi-square statistics; df = degrees of freedom; ΔS-B χ^2 = Satorra–Bentler scaled chi-square difference; Δdf = difference in degrees of freedom, r = robust.
** $p < 0.05$, ** $p < .01$, *** $p \leq .001$.*
