# Supplementary material for: Examining early learners’ perceptions of inclusion: adaptation of the student version of the perceptions of inclusion questionnaire for first- and second-grade students (PIQ-EARLY)
Source: Front Psychol. 2023 Jun 12;14:1181546. doi: 10.3389/fpsyg.2023.1181546 (PMC10291259; doi:10.3389/fpsyg.2023.1181546)
Supplement: Supplementary file 3 [file Table_3.pdf]

Supplementary Material

Examining Early Learners' Perceptions of Inclusion: Adaptation of the Student Version of the Perceptions of Inclusion Questionnaire for First- and Second-Grade Students (PIQ-EARLY)

Sandra Grüter*, Janka Goldan, Carmen L. A. Zurbriggen

*** Correspondence:**

Sandra Grüter:
sandra.grueter@uni-bielefeld.de

Supplementary Table 3

Fit Indices of the CFA Models at T1 and T2 (with estimator WLSMV)

Model	CFI	TLI	RMSEA	SRMR	χ^2	df	ΔS-B χ^2	Δdf
				T1 (N = 379)				
M1	0.885	0.860	0.125	0.119	503.092***	54		
M2	0.929	0.911	0.100	0.096	322.586***	53	49.379***	1
M3	0.994	0.992	0.030	0.045	72.448*	51	71.267***	2
				T2 (N = 572)				
M1	0.848	0.815	0.152	0.127	1086.796***	54		
M2	0.920	0.900	0.112	0.098	595.258***	53	142.38***	1
M3	0.988	0.985	0.044	0.045	120.475***	51	100.89***	2

Note.
CFI = comparative fit index; TLI = Tucker–Lewis index; RMSEA = root mean square error of approximation; SRMR = standardized root mean residual; χ^2= Satorra–Bentler scaled chi-square statistics; df = degrees of freedom; Δ S-B χ^2 = Satorra–Bentler scaled chi-square difference; Δ df = difference in degrees of freedom.
** $p < 0.05$, ** $p < .01$, *** $p \leq .001$.*
