# Supplementary material for: Examining early learners’ perceptions of inclusion: adaptation of the student version of the perceptions of inclusion questionnaire for first- and second-grade students (PIQ-EARLY)
Source: Front Psychol. 2023 Jun 12;14:1181546. doi: 10.3389/fpsyg.2023.1181546 (PMC10291259; doi:10.3389/fpsyg.2023.1181546)
Supplement: Supplementary file 4 [file Table_4.pdf]

Supplementary Material

Examining Early Learners' Perceptions of Inclusion: Adaptation of the Student Version of the Perceptions of Inclusion Questionnaire for First- and Second-Grade Students (PIQ-EARLY)

Sandra Grüter*, Janka Goldan, Carmen L. A. Zurbriggen

*** Correspondence:**

Sandra Grüter:
sandra.grueter@uni-bielefeld.de

Supplementary Table 4

Descriptive Statistics and Standardized Factor Loading of the PIQ Items (T1 data)

Variable	*M*	*SD*	Median	min	max	(1)	(2)	(3)	(4)	Skew	Kurtosis	*N*	λ
Emotional Inclusion													
SW1	3.55	0.75	4	1	4	4.2	3.2	26.1	66.5	-1.87	3.28	403	.81
SW2	3.43	0.88	4	1	4	6.4	6.9	24.2	62.5	-1.51	1.37	405	.82
SW3	3.64	0.74	4	1	4	4.0	3.7	17.1	75.2	-2.23	4.48	404	.75
SW4	3.59	0.76	4	1	4	3.5	6.0	19.0	71.6	-1.91	3.04	401	.75
Scale mean	3.55	0.66	3.75	1.00	4.00	-	-	-	-	-1.90	3.55	406	
Social Inclusion													
SI1	3.60	0.65	4	1	4	1.5	4.7	26.2	67.7	-1.69	2.77	405	.45
SI2	3.52	0.71	4	1	4	1.8	7.5	27.8	62.9	-1.42	1.52	399	.61
SI3	3.69	0.66	4	1	4	2.5	3.7	16.3	77.5	-2.37	5.52	404	.62
SI4	3.48	0.68	4	1	4	1.5	6.2	35.2	57.1	-1.22	1.27	401	.68
Scale mean	3.57	0.49	3.75	1.25	4.00	-	-	-	-	-1.44	2.38	407	
Academic self-concept													
AS1	3.32	0.81	3	1	4	4.5	8.4	38.1	49.0	-1.14	.85	404	.52
AS2	3.24	0.92	3	1	4	7.2	11.4	31.8	49.5	-1.04	0.14	402	.63
AS3	3.74	0.54	4	1	4	1.0	1.7	19.8	77.5	-2.32	6.34	404	.55
AS4	3.55	0.77	4	1	4	3.5	6.9	21.1	68.5	-1.74	2.35	403	.65
Scale mean	3.45	0.55	3.5	1.00	4.00	-	-	-	-	-1.14	1.18	407	

Note. 1 = *not at all true*, 2 = *rather not true*, 3 = *somewhat true*, 4 = *certainly true*
