# Supplementary material for: Examining early learners’ perceptions of inclusion: adaptation of the student version of the perceptions of inclusion questionnaire for first- and second-grade students (PIQ-EARLY)
Source: Front Psychol. 2023 Jun 12;14:1181546. doi: 10.3389/fpsyg.2023.1181546 (PMC10291259; doi:10.3389/fpsyg.2023.1181546)
Supplement: Supplementary file 5 [file Table_5.pdf]

Supplementary Material

Examining Early Learners' Perceptions of Inclusion: Adaptation of the Student Version of the Perceptions of Inclusion Questionnaire for First- and Second-Grade Students (PIQ-EARLY)

Sandra Grüter*, Janka Goldan, Carmen L. A. Zurbriggen

*** Correspondence:**

Sandra Grüter:
sandra.grueter@uni-bielefeld.de

Supplementary Table 5

Descriptive Statistics and Standardized Factor Loading of the PIQ Items (T2 data)

Variable	*M*	*SD*	Median	min	max	(1)	(2)	(3)	(4)	Skew	Kurtosis	*N*	λ
Emotional Inclusion													
SW1	3.44	0.77	4	1	4	3.9	5.8	32.6	57.7	-1.45	1.78	608	.78
SW2	3.28	0.90	4	1	4	6.3	11.4	30.0	52.3	-1.10	0.30	606	.81
SW3	3.58	0.74	4	1	4	3.3	5.1	21.7	69.9	-1.90	3.18	605	.77
SW4	3.47	0.83	4	1	4	4.9	6.9	24.1	64.1	-1.59	1.77	610	.73
Scale mean	3.44	0.68	3.75	1	4	-	-	-	-	-.143	1.83	611	
Social Inclusion													
SI1	3.54	0.71	4	1	4	2.1	6.3	26.7	64.9	-1.58	2.15	606	.52
SI2	3.50	0.71	4	1	4	2.0	6.4	31.2	60.4	-1.39	1.66	608	.65
SI3	3.63	0.67	4	1	4	2.3	4.0	22.6	71.1	-1.99	3.94	606	.73
SI4	3.40	0.77	4	1	4	2.5	9.9	32.2	55.4	-1.16	0.73	605	.70
Scale mean	3.52	0.54	3.75	1	4	-	-	-	-	-1.50	2.70	613	
Academic self-concept													
AS1	3.33	0.83	4	1	4	4.9	8.7	35.0	51.3	-1.19	0.82	608	.70
AS2	3.20	0.96	3	1	4	9.2	10.4	32.0	48.4	-1.03	0.02	607	.68
AS3	3.68	0.62	4	1	4	1.8	3.0	20.8	74.5	-2.2	5.16	607	.53
AS4	3.56	0.76	4	1	4	4.0	4.8	22.2	69.0	-1.88	3.07	607	.59
Scale mean	3.44	0.59	3.5	1	4	-	-	-	-	-1.26	1.58	612	

Note. 1 = *not at all true*, 2 = *rather not true*, 3 = *somewhat true*, 4 = *certainly true*
